# Supplementary material for: Systemic Influences of Mammary Cancer on Monocytes in Mice
Source: Cancers (Basel). 2022 Feb 7;14(3):833. doi: 10.3390/cancers14030833 (PMC8834227; doi:10.3390/cancers14030833)

Supplementary Figure Legends

Supplementary Figure S1: (A) Flow cytometric gating and cell-sorting strategy for blood monocytes and neutrophils used in Figure 1, 2, 4 and 6. (B) Total number of cells in each monocyte subpopulation in 50µl of blood from mice with ~20mm tumour late-cancer (red dots) versus controls (black dots). * p value <0.05, ** p value <0.01, *** p value <0.001, multiple t-test. Data is combined from two independent experiments, with n=12 (cancer) and n=13 (control) mice in total.

Supplementary Figure S2: Flow cytometric gating strategy for BM monocytes in Figure 1 and Figure 2E.

Supplementary Figure S3: Flow cytometric gating strategy for identification and sorting of BM Ly6C^{high} monocytes, MDPs and cMoPs in Figure 2A, C and Figure 6.

Supplementary Figure S4: Flow cytometric gating strategy for identification and sorting of BM LK in Figure 2A and B.

Supplementary Figure S5: Flow cytometric gating strategy for identification of BM myeloid progenitors in Figure 2D.

Supplementary Figure S6: Flow cytometric gating strategy for identification of splenic Ly6C^{high} monocytes and MDP in Figure 3.

Supplementary Figure S7: Identification of BrdU^{+} monocytes in the blood (A), BM (B) and spleen (C) in Figure 2D-F and Figure 3F. Example staining taken at 24 (A) or 1 (B-C) hrs post-injection of BrdU.

Supplementary Figure S 8: Bulk RNA sequencing of Ly6C^{high} and Ly6C^{low} blood monocytes from C57BL/6 mice with late cancer and littermate controls. *(A) PCA of all*

monocytic samples derived from total RNAseq. (B) PCA of Ly6C^{low} monocytes derived from total RNAseq.

Supplementary Figure S1

A

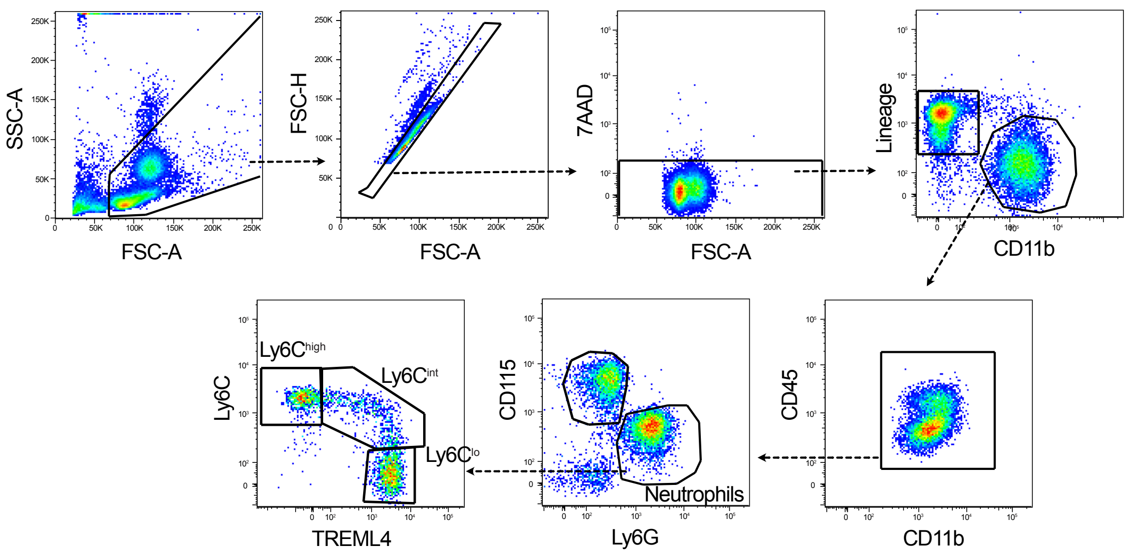

B

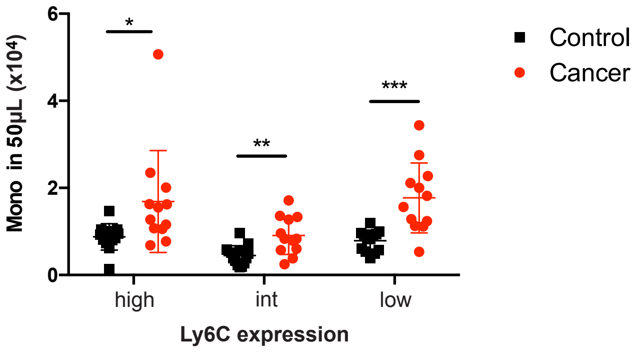

Supplementary Figure S2

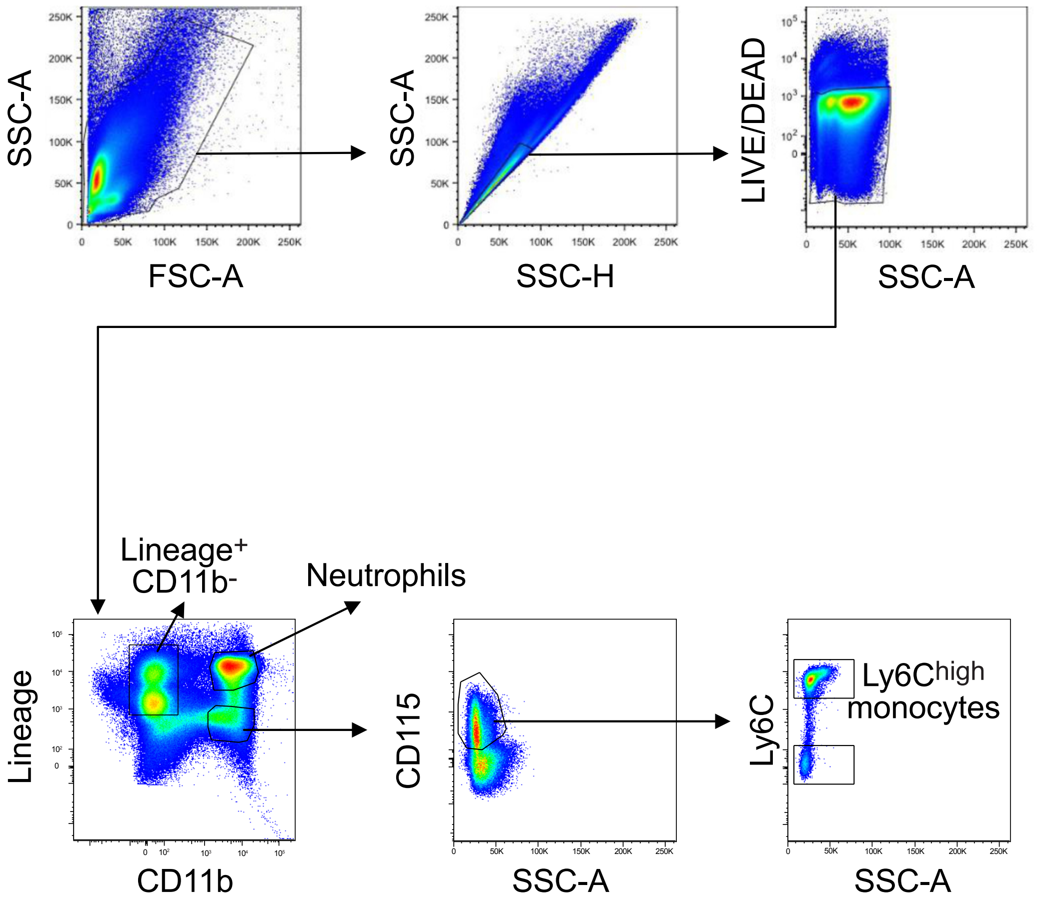

Supplementary Figure S3

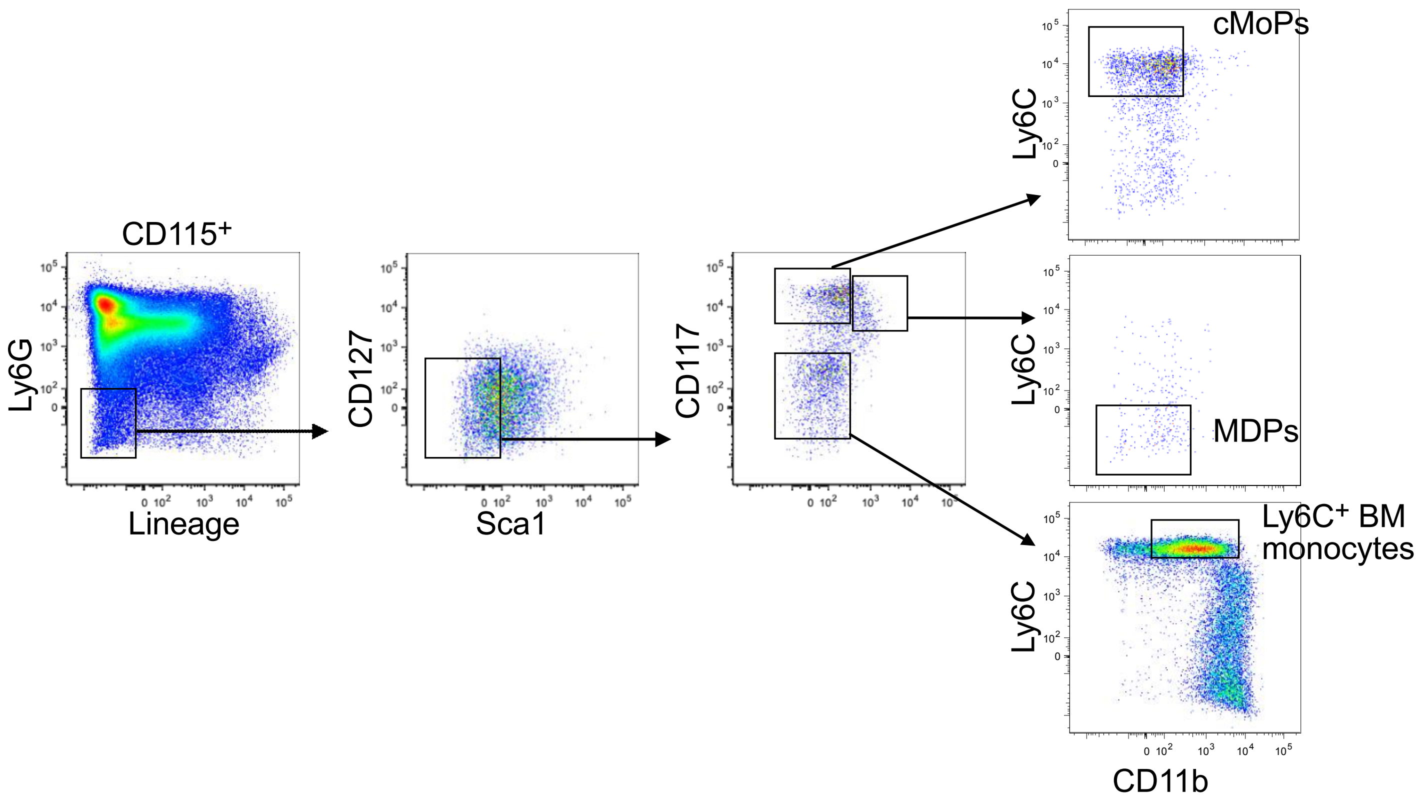

Supplementary Figure S4

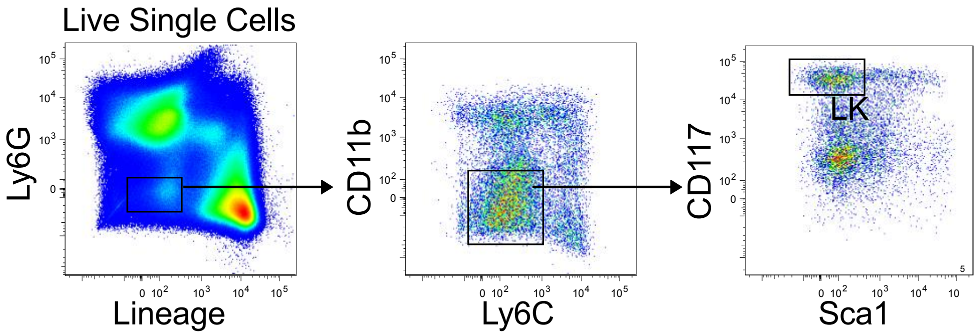

Supplementary Figure S5

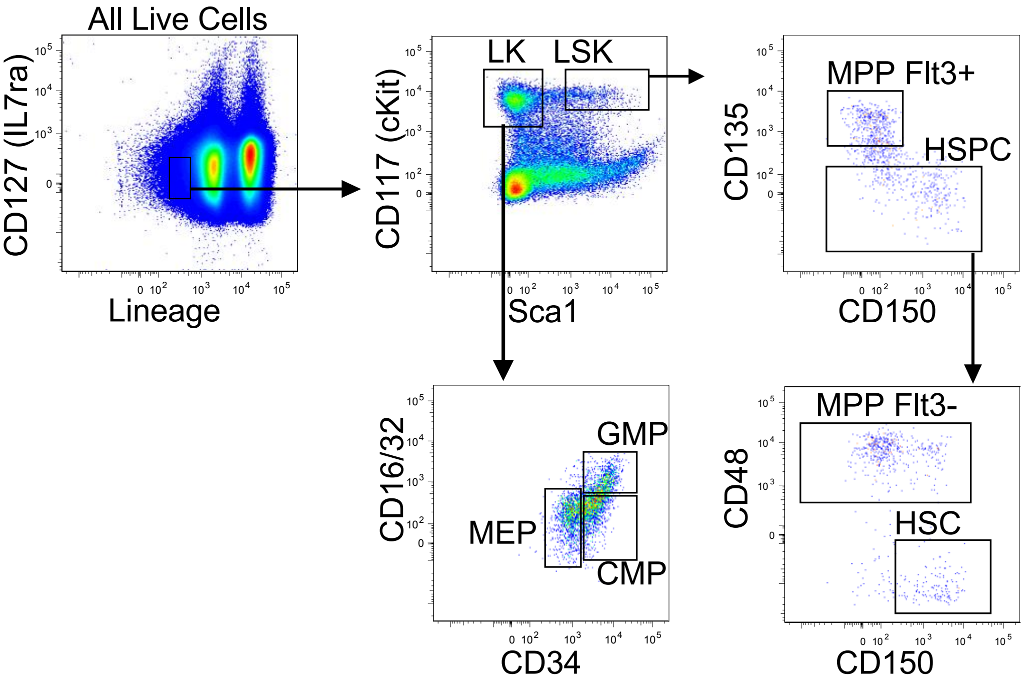

Supplementary Figure S6

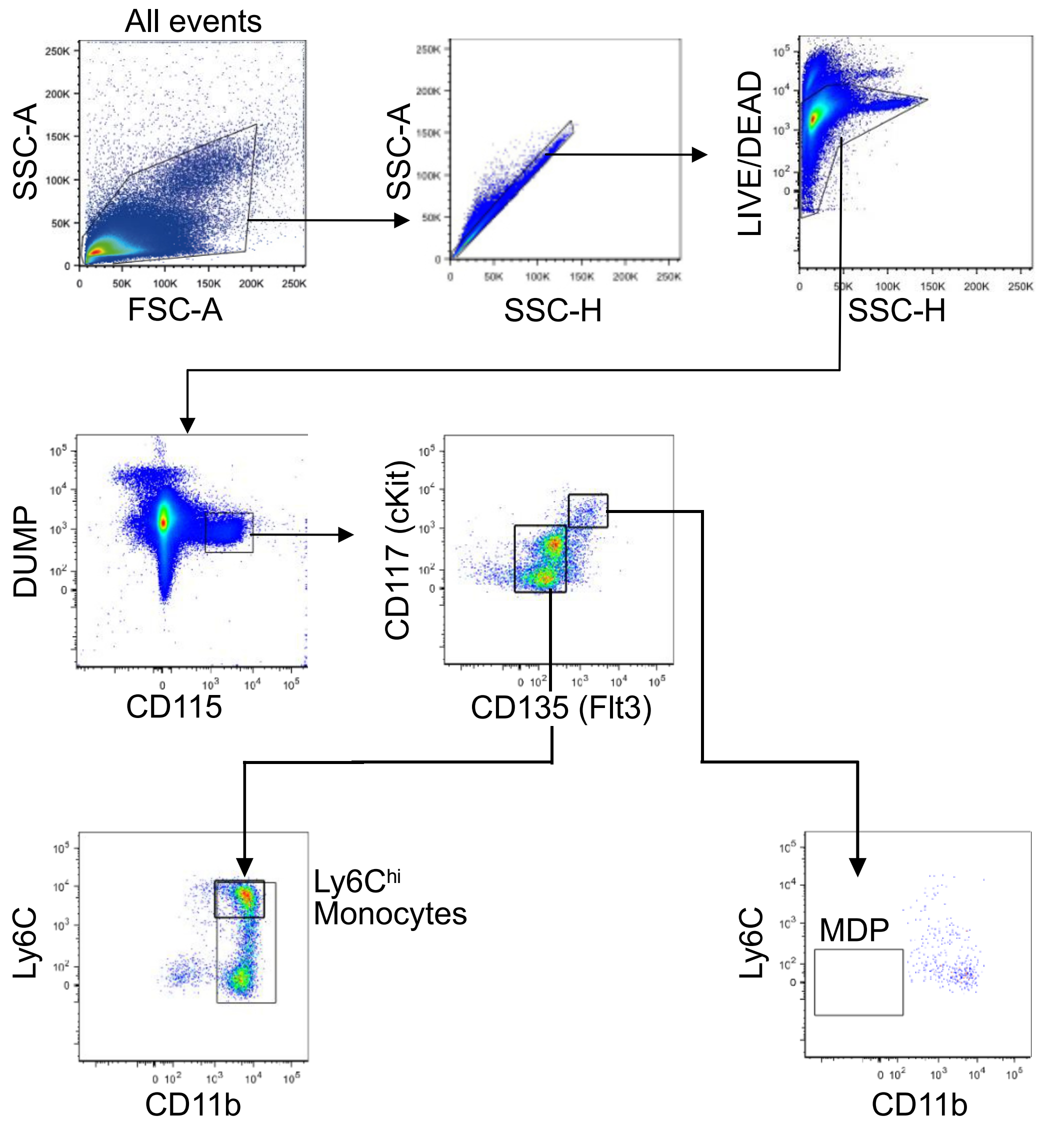

Supplementary Figure S7

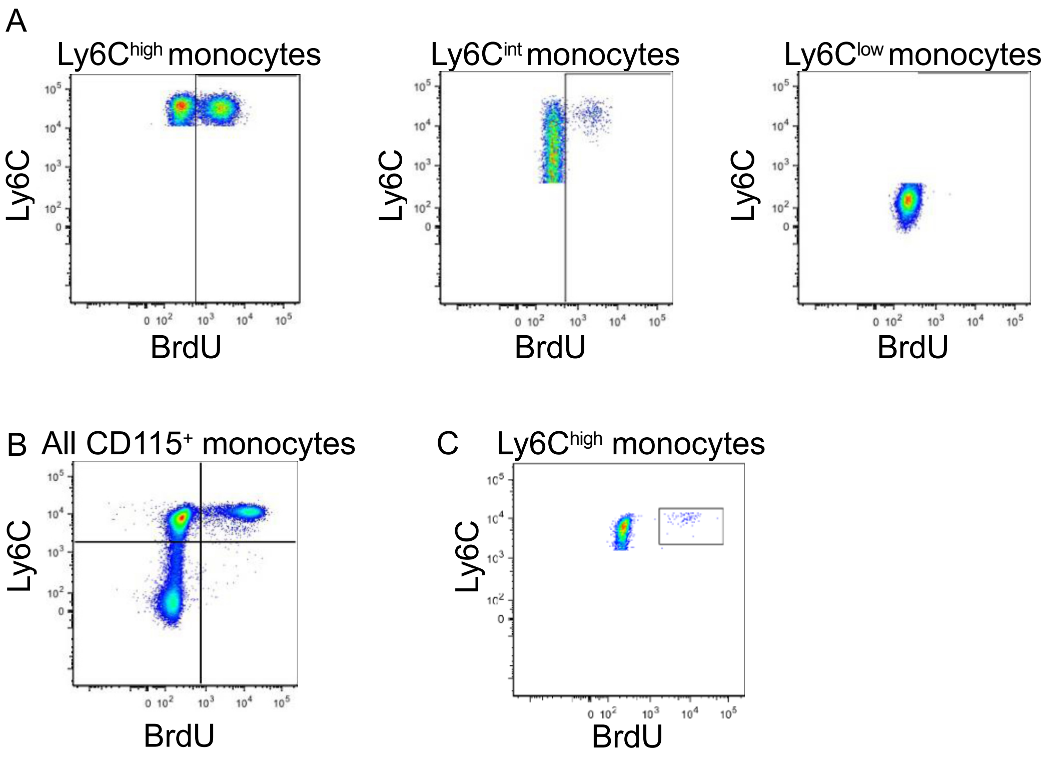

Supplementary Figure S8

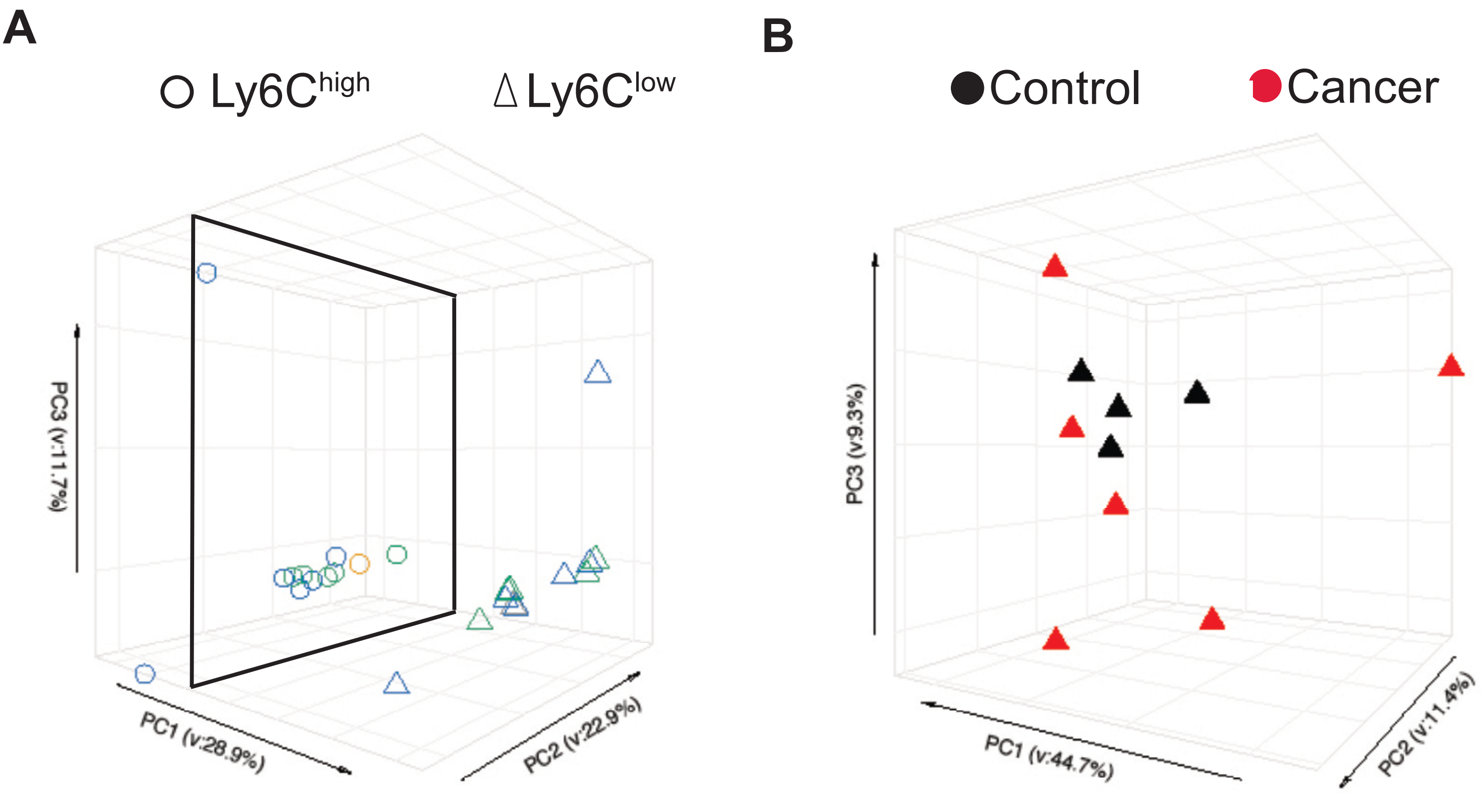

Supplement: Supplementary file 1 [file cancers-14-00833-s001.zip › Suplementary figures and legends.pdf]
